# Retraction Notice to: Overexpression of microRNA-203 Suppresses Proliferation, Invasion, and Migration while Accelerating Apoptosis of CSCC Cell Line SCL-1

**DOI:** 10.1016/j.omtn.2022.05.002

**Published:** 2022-05-10

**Authors:** Wenyun Ting, Cheng Feng, Mingzi Zhang, Fei Long, Ming Bai

## Main Text

(Molecular Therapy: Nucleic Acids *21*, 428–440; September 2020)

This article has been retracted at the request of the editors of *Molecular Therapy – Nucleic Acids*.

After investigating concerns raised by a reader, evidence for misrepresentation of patient survival data in Figure 1B was identified. This misrepresentation of data without appropriate attribution represents a severe abuse of the scientific publishing system. The authors did not respond to the request to retract the paper.

